# Relationships between intrauterine fetal growth trajectories and markers of adiposity and inflammation in young adults

**DOI:** 10.1038/s41366-022-01203-2

**Published:** 2022-08-17

**Authors:** Ashish Yadav, Lawrence J. Beilin, Rae-Chi Huang, Phil Vlaskovsky, John P. Newnham, Scott W. White, Trevor A. Mori

**Affiliations:** 1 Medical School, University of Western Australia, Perth, WA Australia; 2 Nutrition Health Innovation Research Institute, Edith Cowan University, Perth, WA Australia; 3 Division of Obstetrics and Gynaecology, University of Western Australia, Perth, WA Australia

**Keywords:** Epidemiology, Risk factors, Cardiovascular diseases

## Abstract

**Background:**

There is now good evidence that events during gestation significantly influence the developmental well-being of an individual in later life. This study aimed to investigate the relationships between intrauterine growth trajectories determined by serial ultrasound and subsequent markers of adiposity and inflammation in the 27-year-old adult offspring from the Raine Study, an Australian longitudinal pregnancy cohort.

**Methods:**

Ultrasound fetal biometric measurements including abdominal circumference (AC), femur length (FL), and head circumference (HC) from 1333 mother-fetal pairs (Gen1–Gen2) in the Raine Study were used to develop fetal growth trajectories using group-based trajectory modeling. Linear mixed modeling investigated the relationship between adult body mass index (BMI), waist circumference (WC), and high-sensitivity C-reactive protein (hs-CRP) of Gen2 at 20 (*n* = 485), 22 (*n* = 421) and 27 (*n* = 437) years and the fetal growth trajectory groups, adjusting for age, sex, adult lifestyle factors, and maternal factors during pregnancy.

**Results:**

Seven AC, five FL and five HC growth trajectory groups were identified. Compared to the average-stable (reference) group, a lower adult BMI was observed in two falling AC trajectories: (*β* = −1.45 kg/m^2^, 95% CI: −2.43 to −0.46, *P* = 0.004) and (*β* = −1.01 kg/m^2^, 95% CI: −1.96 to −0.05, *P* = 0.038). Conversely, higher adult BMI (2.58 kg/m^2^, 95% CI: 0.98 to 4.18, *P* = 0.002) and hs-CRP (37%, 95% CI: 9–73%, *P* = 0.008) were observed in a rising FL trajectory compared to the reference group. A high-stable HC trajectory associated with 20% lower adult hs-CRP (95% CI: 5–33%, *P* = 0.011).

**Conclusion:**

This study highlights the importance of understanding causes of the unique patterns of intrauterine growth. Different fetal growth trajectories from early pregnancy associate with subsequent adult adiposity and inflammation, which predispose to the risk of diabetes and cardiometabolic disease.

## Introduction

Obesity has become a major health problem, with approximately half of the adults worldwide either overweight or obese in 2016 [1]. Recent data show 53% adults of the European Union (2019) [2] and 64% from the USA (2017–18) [3] are either overweight or obese, whilst the prevalence among Australians has increased from 57% in 1995 to 67% in 2017–18 [4]. The increasing burden of obesity presents a significant health challenge; clustering of other atherogenic risk factors including insulin resistance, dyslipidemia, and hypertension predispose to cardiovascular disease, diabetes, fatty liver, and premature mortality, while other disabilities include impaired quality of life, reduced mobility, and mental health disorders [5, 6]. Along with obesity, chronic low-grade inflammation has also been postulated in the development and progression of cardiometabolic disease [7, 8]. High-sensitivity C-reactive protein (hs-CRP), an acute phase reactant secreted from the liver, has been shown to be consistently associated with atherosclerosis [9, 10].

Many genetic and lifestyle factors contribute to the development of obesity and cardiometabolic diseases. Early life determinants may have an important role in programming metabolic control mechanisms including fetal growth in utero and subsequently contribute to cardiometabolic risk in the offspring [11, 12]. Studies of the association between birthweight and adiposity in children and young adults have shown inconsistent results. Although a number of studies have shown a positive linear relationship between birthweight and subsequent obesity [13–16], very-low birthweight has also been associated with higher risk of future adiposity, particularly in preterm babies and those with central adiposity [17–19]. Others have reported a J- or U-shaped relationship, with higher prevalence of obesity seen with low as well as high birthweight [20–23].

The role of environmental influences during critical periods of growth and development on long-term health, including obesity, was demonstrated by Barker et al in the 1990s [24, 25]. Although birthweight is routinely used as a surrogate for antenatal growth, it does not represent a measure of different fetal growth patterns in utero. Studies of the association between antenatal ultrasound measures of fetal growth and subsequent obesity [26–29] have largely relied on fetal anthropometry or estimated fetal weight during one or more trimester, but none has examined patterns of fetal growth throughout gestation.

We have recently reported different intrauterine growth trajectories derived from ultrasounds from 15 weeks gestation to birth in the Raine Study, an Australian longitudinal pregnancy cohort [30]. In particular, restricted fetal head and abdominal circumference associated with higher adult blood pressure independent of a range of confounders, including adult adiposity. As the specific influence of in utero fetal growth patterns on adult adiposity remains unclear, the present study extends our recent findings to investigate the relationships between intrauterine growth trajectories with markers of anthropometry and inflammation in the young adult offspring from the Raine Study.

## Methods

### Study population

The Raine Study is an ongoing multigenerational prospective cohort study that aimed to recruit 2900 pregnant women between 16–18 weeks of pregnancy from King Edward Memorial Hospital and nearby clinics in Perth, Western Australia from May 1989 to November 1991. The study investigated the effect of ultrasound imaging on pregnancy outcomes, as described previously [31]. Pregnant women (Gen1) were randomized to an intervention group that recommended ultrasound imaging at 5 timepoints (18, 24, 28, 34, and 38 weeks gestation) or a control group with ultrasound imaging only at 18 weeks unless clinically warranted. The mothers delivered 2868 live infants (Gen2) who have been followed up prospectively from birth till 27 years. The current analysis uses Gen2 information available from questionnaires, clinical assessments, and biochemistry at 20, 22, and 27 years. Written informed consent was provided by the pregnant women (Gen1) at recruitment and the adult offspring (Gen2) at 20, 22, and 27 years. The study was approved by the Human Research Ethics Committees at King Edward Memorial Hospital and The University of Western Australia.

### Gen1 demographic and lifestyle measures during pregnancy

Self-reported questionnaires at 16 and 34 weeks gestation provided information on Gen1 maternal and paternal socio-demographic characteristics, including ethnicity, marital status, family income, smoking and alcohol drinking. Family income was assessed by annual family income at the time of the first ultrasound scan, low income being < $24 000 (AUS) in 1989–1991. Pregnancy characteristics including maternal weight and height were obtained from maternal medical records. Gestational age was determined from the date of the last menstrual period or by ultrasound estimation at 18 weeks. As gestational weight gain in the 2nd and 3rd trimester associates with fetal growth and birthweight [32, 33], maternal body mass index (BMI) was calculated at 16 weeks and weight gain during pregnancy was calculated between 16 and 34 weeks gestation. Maternal smoking and alcohol drinking were recorded as dichotomous responses, with a positive response suggesting consumption either at 16 or 34 weeks or both. Gestational diabetes was self-reported and recorded by midwives 2 days post-delivery. Preterm pregnancy was defined as live birth at <37 completed weeks. Birthweight and length of the offspring were extracted from hospital records. Hypertension (HTN) in pregnancy was categorized as Uncomplicated-HTN or Complicated-HTN. Mothers with Uncomplicated-HTN had either a history of pre-pregnancy HTN or those who developed HTN during pregnancy but without any evidence of proteinuria or preterm delivery. Complicated-HTN included mothers who developed HTN during pregnancy plus proteinuria (>2+ on dipstix) or 300 mg on 24-h urinary protein excretion or preterm delivery at less than 37 weeks gestation [34]. HTN during pregnancy was defined as any recording of systolic blood pressure (SBP) > 140 mmHg and/or diastolic blood pressure (DBP) > 90 mmHg [35].

### Adult offspring (Gen2) anthropometry & hs-CRP

Body weight was measured using Wedderburn Chair Scales (to the nearest 100 g) with participants dressed in light clothes and height using a Stadiometer (to the nearest 0.1 cm). Waist circumference (WC) was recorded (to the nearest 0.1 cm) using a measuring tape at the halfway point between the lowest rib and the iliac crest. hs-CRP was measured by an immunoturbidimetric method on an Architect c16000 Analyser (CRP Vario test, Abbott Laboratories Inc., IL, USA) (inter-assay CV 2.07%) in the PathWest Laboratory at Royal Perth Hospital. hs-CRP values >10 mg/L at any follow-up were excluded from the analysis if a participant had a BMI < 30 kg/m^2^ across the three follow-ups. Elevated hs-CRP in the absence of obesity is likely to indicate acute inflammation [36].

### Adult offspring (Gen2) demographic and lifestyle measures

Computer-based self-assessment questionnaires were used to assess demographic and socio-behavioral data at 20, 22, and 27 years. Smoking was coded as a dichotomous variable and a participant was considered a smoker if they had smoked a cigarette in the past 1 month. Alcohol consumption was coded as a continuous variable and reported as total ethanol consumption in g/week, with 1 standard drink equivalent to 10 g ethanol. Alcohol intake included the type and amount of alcoholic beverages consumed daily over the past 7 days. Hormonal contraceptive use in females was based on current use of any hormonal contraceptive pill, injection, implants or intrauterine device. Socioeconomic status (SES) of the Gen2 participants was assessed using Socioeconomic indexes for areas (SEIFA) scores, which has been used in Australia and in the Raine Study to quantify SES [37]. Educational status had 3-categories- participants completing high school (Year-12, 17 years of age in Australia); participants with apprenticeship or vocational training; and those in university (tertiary education). Physical activity was measured as metabolic equivalents (MET) and coded as a continuous variable as MET-minutes-per-week where one MET is equal to the amount of oxygen consumed during rest (3.5 ml/kg/min). BMI was calculated as body weight/height^2^ (kg/m^2^) and coded as a continuous variable. World Health Organisation (WHO) criteria were used to define overweight (BMI ≥25 kg/m^2^) and obese (BMI ≥30 kg/m^2^) when categorizing BMI [38].

## Statistical analysis

### Antenatal data and fetal growth trajectories

**Fig. 1:**
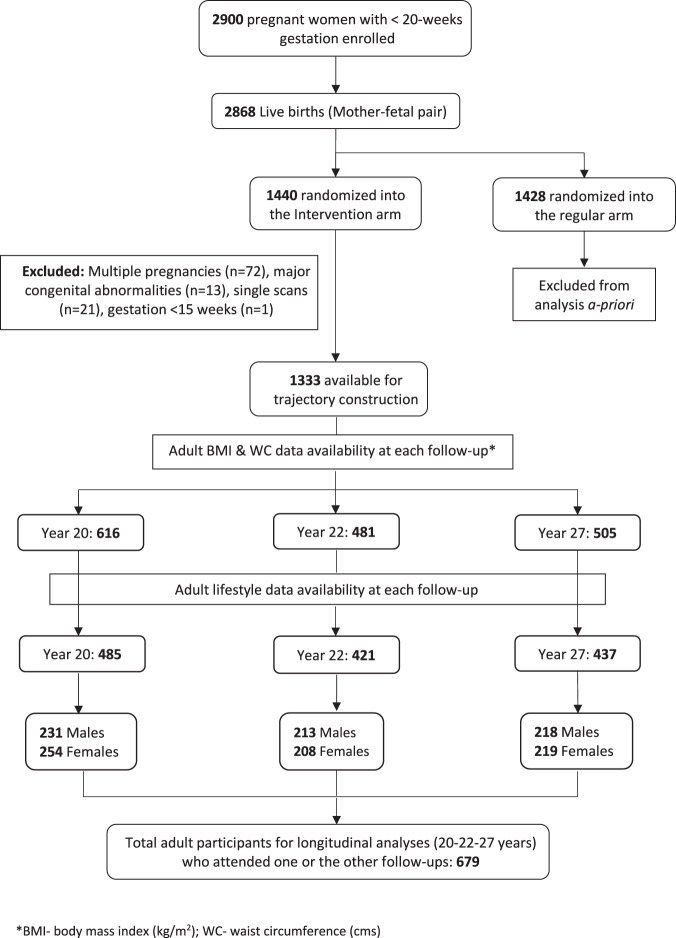
Flow diagram of Raine Study participants attending the 20, 22 and 27-year follow-up. Flow diagram of Raine Study participants attending the 20, 22 and 27-year follow-up with completeadult body mass index (BMI), waist circumference (WC) and lifestyle data.

**Fig. 2:**
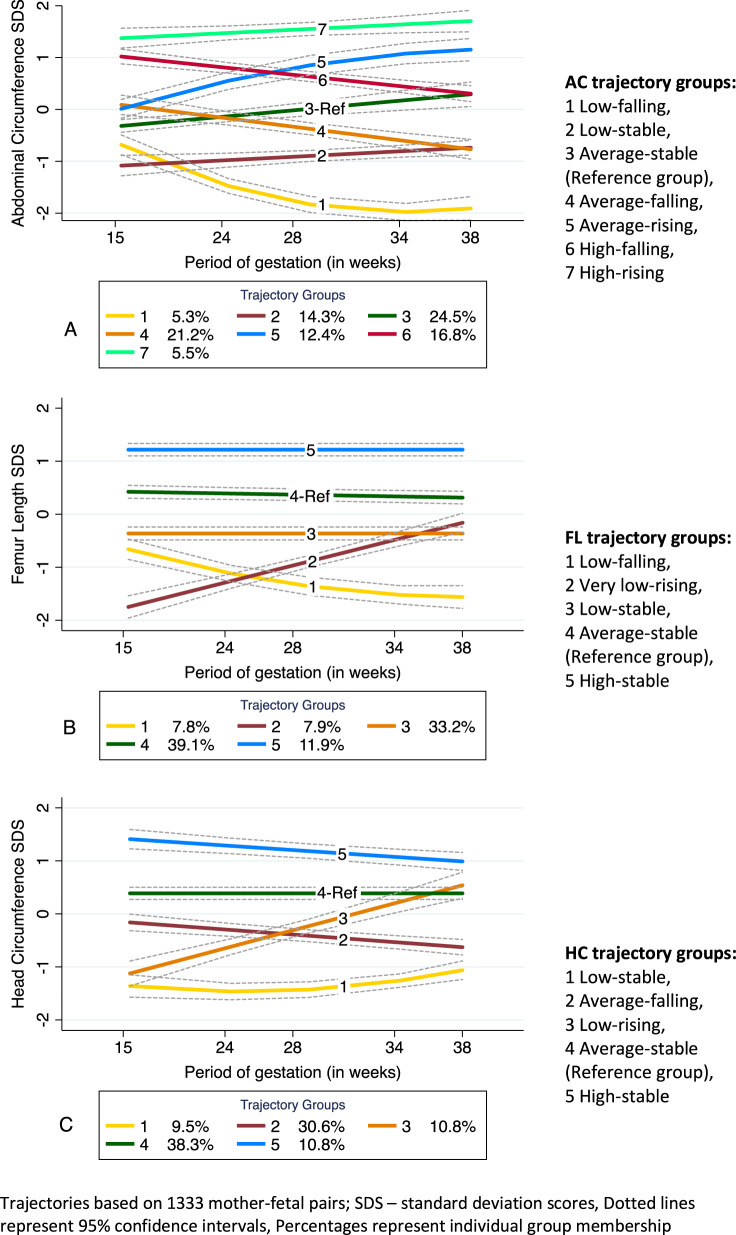
Fetal growth trajectories using abdominal circumference (AC), femur length (FL), and head circumference (HC) anthropometric markers. Reproduced from Yadav et al. [30]. DOI:10.1097/HJH.0000000000003035 with permission from Wolters Kluwer Health, Inc.

Ultrasound measurements of fetal abdominal circumference (AC), femur length (FL) and head circumference (HC) were used to construct fetal growth trajectories from 1333 mother-fetal pairs, after necessary exclusions (Fig. 1). Details relating to trajectory construction have been published [30]. In brief, standard deviation scores (SDS) were calculated for ultrasound-based fetal anthropometric markers using linear regression, adjusting for physiological factors influencing fetal growth (maternal height and parity, sex, and ethnicity of the fetus). To facilitate trajectory development using a minimum of two data points and to eliminate selection bias, ultrasound measurements from only the intervention arm of the randomized controlled trial were chosen for analysis. Maternal height was centered at 164 cm (average height of women during 1980–90s) and gestational age at 28 weeks (196 days, mean gestational age). Ethnicity of the fetus/offspring was dichotomized and categorized as Caucasian if both parents were Caucasians. Using a Stata plug-in, group-based trajectory modeling (GBTM) was applied on each fetal anthropometric marker SDS and seven AC, five FL, and five HC trajectory groups were identified (Fig. 2).

### Effect of trajectories on Gen2 adult anthropometry and hs-CRP

Random coefficient linear mixed modeling (LMM) was used to estimate the effects of trajectory groups on adult BMI, WC and hs-CRP at 20, 22, and 27 years assessed together. Bootstrapping with 500 repetitions was performed to attenuate the potential influence of outlier observations and provide more robust estimates of parameter estimates’ error. Data for fetal growth trajectories and Gen2 participants’ lifestyle factors were available for 485, 421, and 437 adults at 20, 22, and 27 years, respectively, representing 679 individuals attending one or more follow-up (Fig. 1). Non-normality of the outcome variables was accounted for using linear mixed regression modeling and further by bootstrapping [39, 40]. hs-CRP was log-transformed before bootstrapping due to the presence of significant skewing. The model residuals were subsequently checked to confirm normal distribution. The selection of confounders was determined a priori and was based on scientific evidence, statistical reasoning, and data availability. Adult covariates included age at follow-up, sex, female contraceptive use, ethnicity, alcohol intake, smoking, adult SES, educational status, and physical activity for BMI and WC analysis, plus BMI for hs-CRP analysis. Maternal covariates included family income, smoking, alcohol drinking, weight gain during pregnancy, maternal BMI, preterm pregnancy, gestational diabetes mellitus (GDM), Uncomplicated-HTN, Complicated-HTN and breast feeding >6 months. Univariate analysis using a P-value cut-off of 0.1 was applied to select variables for multivariate modeling. To select the most parsimonious model, stepwise backwards selection was performed based on a P-value threshold as well as change in estimate, both approaches carried out manually to determine the final set of confounders. A very conservative approach was followed by relying on a significant global P-value for the trajectory group variable along with local P-values for each group. Age, sex, contraceptive use in females, SES, and ethnicity of Gen2 adults were included in all multivariate models. Final covariates for Model 1 included age, sex, female contraceptive use, ethnicity, SES and physical activity. Model 2 additionally accounted for the pregnancy covariates namely uncomplicated hypertension during pregnancy, maternal alcohol drinking, and maternal BMI at 16 weeks. All models with hs-CRP as the outcome variable adjusted for BMI. BMI was centered at 25 kg/m^2^ and modeled with a second-order polynomial. Gen2 physical activity and SES variables were normalized in the multivariate modeling using z-score standardization. Interaction between trajectory groups and sex of Gen2 adults was explored for all models. Birthweight of Gen2 adults was also analyzed as a continuous variable to estimate the effect on adiposity measures.

Results are presented as percentages, means with standard deviations or medians with upper and lower quartiles. Stata v17.0 (Stata Corp., College Station, TX, USA) was used for all statistical analyses with two-sided significance set at *P* < 0.05. Stata codes are available for researchers upon request to authors.

## Results

Table 1 shows the general characteristics of the participants (Gen2) at 20, 22, and 27 years and their mothers (Gen1) during pregnancy. Approximately 90% of Gen2 were Caucasians. Males and females had a similar BMI at all three ages. However, males were more likely to be overweight (BMI > 25 and <30 kg/m^2^) and have a greater waist circumference, and more females had obesity (BMI > 30 kg/m^2^) at each age. Median hs-CRP values tended to be higher in females compared to males at each follow-up. Alcohol was consumed by approximately 70–85% of males and females at 20, 22, and 27 years. The frequency of smoking was ~16% at 20-years and ~22% at 27 years. Physical activity in males and females was higher at 20-years than at 22 and 27 years. Female hormonal contraceptive use was 60.2% at 20 years and 48.9% at 27 years. Birthweights were similar between males and females. In relation to maternal characteristics (Gen1), 7% reported preterm deliveries, 3.3% gestational diabetes, 26.4% Uncomplicated-HTN and 4.7% complicated HTN (Table 1). More than 16% of Gen1 mothers smoked and >56% consumed alcohol during pregnancy.

**Table 1:** General characteristics of the participants (Gen2) at 20, 22 and 27 years and their mothers (Gen1) during pregnancy.

Characteristics	Year 20 (*n* = 485)		Year 22 (*n* = 421)		Year 27 (*n* = 437)	
	Males *n* = 231	Females *n* = 254	Males *n* = 213	Females *n* = 208	Males *n* = 218	Females *n* = 219
**Adult participants (Gen2)**						
Age (years) Mean (SD)	20.1 (0.4)	20.1 (0.4)	22.2 (0.8)	22.2 (0.8)	26.7 (0.4)	26.8 (0.4)
BMI (kg/m^2^) Median (Q1, Q3)	23.5 (21.1,26.4)	23.1 (20.9,26.4)	24.2 (22.1,27.2)	23.8 (21.5,28.0)	24.7 (22.6,27.6)	23.7 (22.6,27.6)
*BMI categories						
<25 (kg/m^2^)	64.5	67.7	60.6	61.5	51.4	61.6
Overweight	23.4	17.7	28.6	17.8	34.4	20.1
Obese	12.1	14.6	10.8	20.7	14.2	18.3
Waist circumference (cm) Median (Q1, Q3)	79.1 (74.8,87.8)	73.9 (68.0,82.2)	83.5 (77.0,91.0)	76.0 (70.5,89.5)	85.5 (79.8,94.3)	76.6 (71.1,85.5)
hs-CRP (mg/L) Median (Q1, Q3)	0.71 (1.2)	1.38 (2.8)	0.65 (1.0)	1.53 (4.2)	0.70 (1.4)	1.10 (2.6)
Alcohol drinkers (%)	75.3	70.1	85.0	79.8	74.8	69.9
Alcohol intake (g/wk ethanol) Median (Q1, Q3)	80.0 (10.0,90.0)	50.0 (0.0,120.0)	109.7 (39.8,240.3)	50.0 (11.1,103.5)	79.2 (0.0,202.5)	42.2 (0.0,118.8)
Smoking (% smokers)	15.6	16.1	17.4	16.4	23.4	21.0
Physical Activity (METmin /week) Median (Q1, Q3)	7386.0 (3546.0, 13050.0)	9936.0 (3336.0, 16398.0)	3816.0 (1893.0, 7164.0)	2147.5 (801.0, 4563.0)	2698.5 (1200.0, 5112.0)	1920.0 (495.0, 3564.0)
Socioeconomic status (SEIFA score) Median (Q1, Q3)	1072.8 (995.7, 1114.4)	1075.9 (1006.8, 1121.5)	1080.9 (1002.9, 1120.0)	1069.0 (1006.8, 1116.5)	1079.1 (1003.9, 1118.4)	1069.3 (1006.8, 1116.5)
Educational status (%)						
Category-1	81.6	72.4	52.4	51.5	21.5	19.6
Category-2	15.0	20.7	26.0	19.8	32.6	28.0
Category-3	3.4	6.9	21.6	28.7	45.8	52.4
^#^Contraceptive use in females (%)	-	60.2	-	59.6	-	48.9
Ethnicity (% Caucasians)	87.9	89.8	88.7	90.9	89.9	91.3
Breastfed >6 months (%)	57.7	57.9	58.5	58.1	58.8	53.8
Birthweight (g) Mean (SD)	3371.5 (552.3)	3308.8 (510.2)	3343.1 (581.9)	3322.2 (516.5)	3416.1 (572.5)	3338.3 (528.4)
Birth length (cm) Mean (SD)	49.3 (2.7)	48.7 (2.3)	49.1 (3.1)	48.7 (2.2)	49.5 (2.9)	48.8 (2.3)
**Maternal (Gen1)**						
BMI (kg/m^2^) Median (Q1, Q3)	22.9 (21.1,24.8)	23.2 (21.2,25.9)	22.8 (20.7,24.6)	23.5 (21.0,26.2)	22.9(21.1,25.5)	23.4 (21.1,25.9)
Preterm delivery (%)	4.8	5.5	7.0	5.3	6.0	4.1
GDM (%)	2.6	1.6	3.3	1.4	3.2	0
Maternal smoking (%)	21.2	23.2	18.3	24.0	16.5	25.6
Alcohol drinkers (%)	60.6	57.5	59.2	56.3	58.3	58.0
Uncomplicated-HTN (%)	26.4	18.5	25.8	20.7	26.2	19.2
Complicated-HTN (%)	3.5	2.8	4.7	1.0	3.2	2.3
Low income (%)	33.2	34.6	37.3	34.2	38.8	34.4

*SD* standard deviation, *Q1* 1st quartile or 25th percentile, *Q3* 3rd quartile or 75th percentile, *BMI* body mass index, *hs-CRP* high-sensitivity C-reactive protein.

*BMI categories: <25, overweight (≥25 and <30), obese (≥30) [in kg/m^2^]; SEIFA: Socioeconomic indexes for areas; Educational status: Category-1 Those completing high school, Category-2 Those with apprenticeship or vocational training, Category-3 Those in university.

^#^Contraception refers to the use of hormonal contraceptives; GDM- gestational diabetes mellitus; HTN- hypertension; Low income- family income at the time of conception, low being annual income < $24,000 (AUS).

### Abdominal circumference (AC) trajectories

#### Relationship of AC trajectories with BMI

**Fig. 3:**
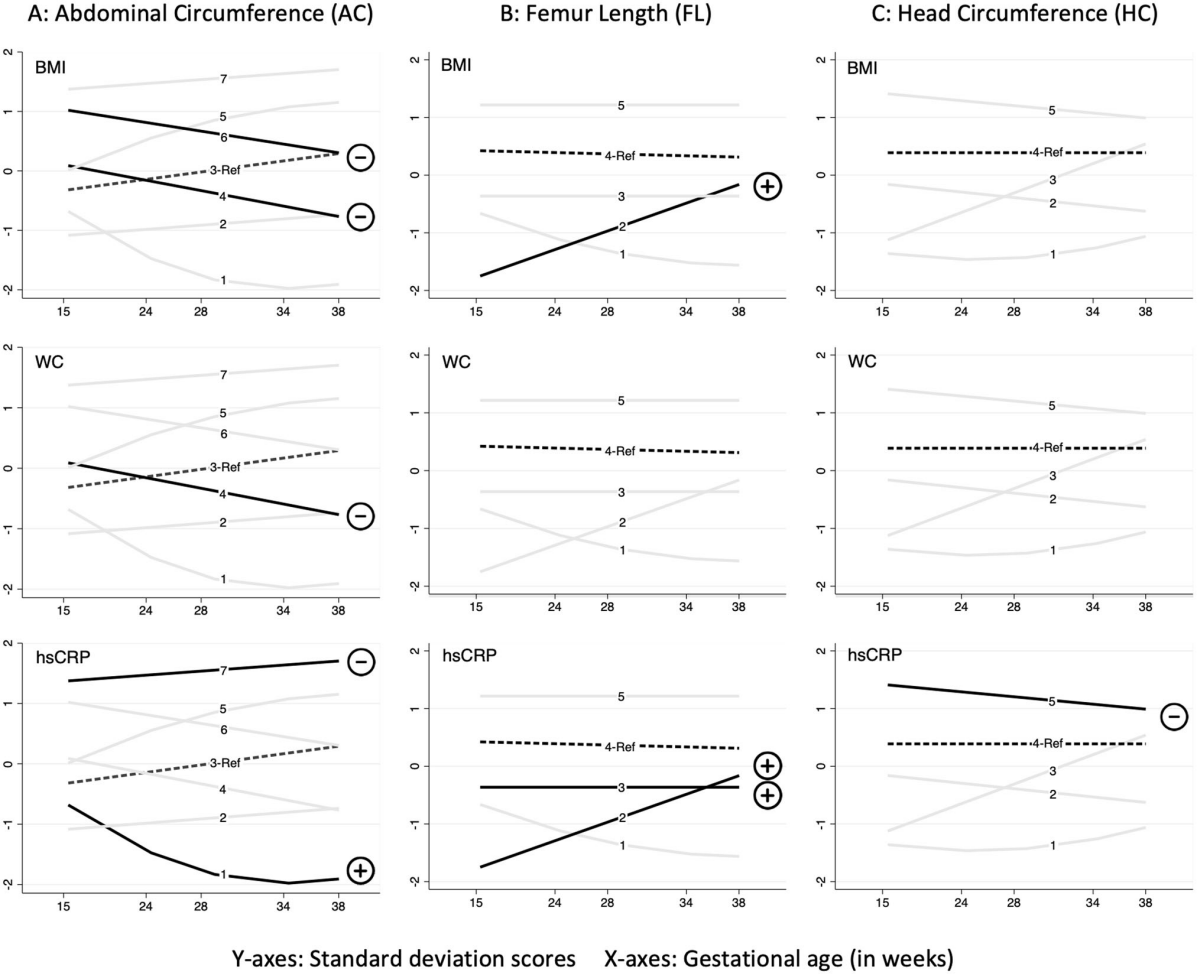
Relationships observed between fetal growth trajectories and adult BMI, WC and hs-CRP. Associations observed from linear mixed modeling (Model 1) between growth trajectories (A-Abdominal circumference, B-Femur Length and C-Head Circumference) and adult outcomes (BMI- Body mass index, WC waistcircumference, hs-CRP- high sensitivity C-reactive protein). Y-axes: Standard deviation scores or z-scores for AC, FL and HC. X-axes: Gestational age in weeks. Dashed lines (--) represent the reference trajectory group while solid-bold lines (–) represent those trajectory groups having a significant association with reference group. Minus sign (−) indicates a negative association while a plus sign (+) indicates a positive association.

Compared with the reference average-stable trajectory (Group-3), participants in Group-4 (average-falling) (*β* = −1.45 kg/m^2^, 95% CI = −2.43, −0.46) and Group-6 (high-falling) (*β* = −1.01 kg/m^2^, 95% CI = −1.96, −0.05) had significantly lower BMI (*P* = 0.004 and *P* = 0.038, respectively) in models that accounted for age, sex, female contraceptive use, ethnicity, SES and physical activity (Table 2A BMI, Model 1 and Supplementary Table 1). The associations persisted for Group-4 when further adjusted for maternal covariates (Table 2A BMI, Model 2 and Supplementary Table 2). Maternal uncomplicated hypertension (*P* = 0.010), maternal alcohol drinking (*P* = 0.006) and maternal BMI at 16 weeks (*P* < 0.001) were independently associated with offspring BMI (Supplementary Table 2). The observed associations between fetal growth trajectory groups for all three anthropometric parameters (AC, FL, and HC) and adult BMI, WC and hs-CRP, based on linear mixed modeling (Model 1) have been summarized in Fig. 3. No significant sex-trajectory interaction was detected.

**Table 2:** Fetal growth trajectories and their association with BMI, WC & hs-CRP.

**2A: Abdominal circumference (AC)**								
^#^Trajectories (Group membership)		Group-1 Low-falling 5.3% (*n* = 36)	Group-2 Low-stable 13.1% (*n* = 89)	Group-4 Average-falling 20.6% (*n* = 139)	Group-5 Average-rising 12.4% (*n* = 84)	Group-6 High-falling 18.3% (*n* = 124)	Group-7 High-rising 5.6% (*n* = 38)	Global *P* value
BMI	Model 1	−0.06	−0.31	−1.45**	0.26	−1.01*	0.20	0.011
	Model 2	0.86	0.21	−1.03*	−0.04	−0.75	−0.47	0.044
WC	Model 1	−0.44	−1.06	−3.10*	0.78	−1.86	0.16	0.033
	Model 2	2.56	0.24	−2.20*	0.20	−1.19	−1.38	0.138
hs-CRP	Model 1	1.40*	1.07	1.05	0.99	0.98	0.70**	0.001
	Model 2	1.48**	1.07	1.06	0.98	0.97	0.70**	0.001

**2B: Femur length (FL)**						
^#^Trajectories (Group membership)		Group-1 Low-falling 5.9% (*n* = 40)	Group-2 Very-low-rising 7.7% (*n* = 52)	Group-3 Low-stable 31.7% (*n* = 215)	Group-5 High-stable 12.4% (*n* = 84)	Global *P* value
BMI	Model 1	0.50	2.58**	0.45	0.35	0.028
	Model 2	0.39	1.77*	0.50	0.05	0.159
WC	Model 1	−1.39	5.22*	0.37	2.04	0.055
	Model 2	−1.62	3.42	0.47	1.29	0.296
hs-CRP	Model 1	1.13	1.37**	1.37***	1.12	<0.001
	Model 2	1.13	1.38**	1.39***	1.12	<0.001

**2C: Head circumference (HC)**						
^#^Trajectories (Group membership)		Group-1 Low-stable 8.0% (*n* = 54)	Group-2 Average-falling 31.5% (*n* = 214)	Group-3 Low-rising 8.1% (*n* = 55)	Group-5 High-stable 11.6% (*n* = 79)	Global *P* value
BMI	Model 1	−0.55	−0.10	0.57	−0.61	0.488
	Model 2	−0.26	−0.37	0.14	−0.82	0.415
WC	Model 1	−0.95	−0.76	1.15	−0.89	0.714
	Model 2	−0.23	−1.33	0.35	−1.34	0.515
hs-CRP	Model 1	1.16	1.05	1.02	0.80*	0.020
	Model 2	1.15	1.04	1.02	0.78**	0.024

Linear mixed modeling results displayed.

*BMI* body mass index (β coefficient in kg/m^2^), *WC* waist circumference (*β* coefficient in cms), *hs-CRP* high-sensitivity C-reactive protein (Exponentiated *β* coefficients).

Significant *P* values: *<0.05, **<0.01, ***<0.001.

^#^Trajectories based on 1333 participants; Reference group is Group-3 (Average-stable) with a membership of 24.9% (*n* = 169) for abdominal circumference, Group-4 (Average-stable) with a membership of 42.4% (*n* = 288) for femur length and Group-4 (Average-stable) with a membership of 40.8% (*n* = 277) for head circumference.

Sample Size: Model 1: BMI and WC- 1242 observations representing 606 participants who attended one or more follow-up; hs-CRP 1139 observations/574 participants. Model 2: BMI and WC- 1223 observations/596 participants; hs-CRP- 1126 observations/566 participants.

Model 1 covariates- age, sex, female contraceptive use, ethnicity, socioeconomic status & physical activity; Model 2 covariates- Model 1 plus uncomplicated hypertension during pregnancy, maternal alcohol drinking, and maternal BMI at 16 weeks (only maternal BMI at 16 weeks for hs-CRP). Models for hs-CRP additionally adjusted for adult BMI.

In analyses examining BMI categories with AC trajectory groups, there were 7.1 and 10.4% participants with obesity in Group-4 and Group-6 respectively, compared to 22.3% in the reference Group-3 at 27 years, with similar trend at 20 and 22 years (Supplementary Table 3).

#### Relationship of AC trajectories with WC

Participants in Group-4 (average-falling) associated with significantly lower WC in Model 1 (*β* = −3.10 cm, 95% CI = −5.48, −0.72) (*P* = 0.011) compared to Group-3 (Table 2A WC, Model 1 and Supplementary Table 1). The association remained significant with further adjustment for maternal covariates (*β* = −2.20 cm, 95% CI = −4.16, −0.24) (*P* = 0.028), although the global P-value was not significant (Table 2A WC, Model 2 and Supplementary Table 2). Maternal covariates independently associated with offspring WC included uncomplicated hypertension (*P* = 0.032), maternal alcohol drinking (*P* = 0.001) and maternal BMI at 16-weeks (*P* < 0.001) (Supplementary Table 2). There was no significant sex-trajectory interaction.

#### Relationship of AC trajectories with hs-CRP

Participants in Group-1 (low-falling) (*β* = 1.40, 95% CI = 1.07, 1.83) had 40% higher (*P* = 0.013) hs-CRP while those in Group-7 (high-rising) (*β* = 0.70, 95% CI = 0.56, 0.88) had 30% lower (*P* = 0.002) hs-CRP, compared with Group-3 (Table 2A hs-CRP, Model 1 and Supplementary Table 1). These associations remained significant when adjusted for maternal covariates (Table 2A hs-CRP, Model 2 and Supplementary Table 2). Maternal BMI at 16 weeks was independently associated with offspring hs-CRP (*P* = 0.036) (Supplementary Table 2). No sex-trajectory interaction was detected.

### Femur length (FL) trajectories

#### Relationship of FL trajectories with BMI & WC

Group-2 (very-low rising) participants associated with significantly higher BMI (*β* = 2.58 kg/m^2^, 95% CI = 0.98, 4.18; *P* = 0.002) in models accounting for age, sex, contraceptive use, ethnicity, SES and physical activity when compared with the reference Group-4 (average-stable) (Table-2B BMI, Model 1 and Supplementary Table 4). Significance for Group-2 was retained (*β* = 1.77 kg/m^2^, 95% CI = 0.20, 3.35; *P* = 0.027) when further adjusted for maternal covariates (Table 2B BMI, Model 2 and Supplementary Table 5). Maternal uncomplicated hypertension (*P* = 0.015), maternal alcohol drinking (*P* = 0.008), and maternal BMI at 16 weeks (*P* < 0.001) were independently associated with offspring BMI (Supplementary Table 5), although with the global *P* value was not significant. No sex-trajectory interaction was detected. 22.2% participants in Group-2 were obese at 27 years compared to 12.6% in the reference Group-4, with similar distribution at 20 and 22 years (Supplementary Table 6). A similar association for Group-2 participants was detected for WC (*β* = 5.22 cm, 95% CI = 1.28, 9.17) (*P* = 0.009), compared to reference Group-4 (Table 2B WC, Model 1 and Supplementary Table 4).

#### Relationship of FL trajectories with hs-CRP

Compared with reference Group-4, participants in both Group-2 (very-low rising) (*β* = 1.37, 95% CI = 1.09, 1.73) and Group-3 (low-stable) (*β* = 1.37, 95% CI = 1.19, 1.58) associated with 37% higher hs-CRP (*P* = 0.008 and *P* < 0.001 respectively), when adjusted for age, sex, contraceptive use, BMI, ethnicity, SES & physical activity (Table 2B hs-CRP, Model 1 and Supplementary Table 4). These associations remained significant with further adjustment for maternal covariates (Table 2B hs-CRP, Model 2 and Supplementary Table 5). Maternal BMI at 16 weeks (*P* = 0.021) was independently associated with adult hs-CRP (Supplementary Table 5). A sex-trajectory interaction was detected (*P* = 0.001) and in sex-specific analysis, males in Group-2 (*β* = 1.74, 95% CI = 1.24, 2.43) had 74% higher (*P* = 0.001) hs-CRP (adjusted for age, BMI, ethnicity, SES and physical activity) while females in Group-3 (β = 1.70, 95% CI = 1.37, 2.11) had 70% higher (*P* < 0.001) hs-CRP (adjusted for age, contraceptive use, BMI, ethnicity, SES and physical activity) (Supplementary Table 7). In both instances, the associations were retained with further adjustment for maternal covariates.

### Head circumference (HC) trajectories

#### Relationship of HC trajectories with BMI & WC

There were no associations of HC growth trajectories with BMI and WC (Table 2C, BMI and WC and Supplementary Tables 8, 9). However, a significant sex-trajectory interaction was detected for BMI (*P* = 0.009) and sex-specific analysis showed females in Group-3 (low-rising) had significantly higher BMI (*β* = 2.52 kg/m^2^, 95% CI = 0.23, 4.81, *P* = 0.031), compared with reference Group-4 (adjusted for age, contraceptive use, ethnicity, SES and physical activity) (Supplementary Table 10). Comparison of BMI categories across HC trajectory groups showed a similar distribution of participants with obesity across groups (Supplementary Table 11).

#### Relationship of HC trajectories with hs-CRP

Participants in Group-5 (high-stable) (*β* = 0.80, 95% CI = 0.67, 0.95) associated with 20% lower hs-CRP (*P* = 0.011), compared to reference Group-4 (average-stable) (Table 2C hs-CRP, Model 1 and Supplementary Table 8) and remained significant with further adjustment for maternal covariates (*β* = 0.78, 95% CI = 0.65, 0.94, *P* = 0.008) Table 2C hs-CRP, Model 2 and Supplementary Table 9. Maternal BMI at 16-weeks (P = 0.026) was independently associated with adult hs-CRP (Supplementary Table 9). There was no sex-trajectory interaction (*P* = 0.294).

### Relationship of birthweight and adult BMI, WC and hs-CRP

Birthweight (kg), corrected for gestational age, was positively associated with BMI (*β* = 1.61 kg/m^2^, 95% CI = 0.79, 2.42, *P* = 0.001) and WC (*β* = 4.62, 95% CI = 2.66, 6.58, *P* < 0.001) after adjusting for adult covariates (Supplementary Table 12, Model 1). The association remained significant with further adjustment for maternal covariates (Supplementary Table 12, Model 2). No significant association of hs-CRP was detected with birthweight (*P* = 0.053).

## Discussion

Using serial ultrasound measures during pregnancy, this study has shown significant relationships between fetal growth patterns and subsequent markers of adiposity and inflammation in 27-year-olds, with some differences between the sexes. The findings show that adult BMI and waist circumference was inversely associated with trajectories reflecting average or above-average abdominal growth from early-mid pregnancy and decelerating. Restricted abdominal circumference throughout pregnancy associated with higher adult hs-CRP, whereas greater-than-average fetal abdominal and head circumference throughout pregnancy was associated with lower hs-CRP in adulthood. The associations between fetal growth and adult adiposity and hs-CRP, largely persisted after adjustment for postnatal lifestyle factors as well as maternal and pregnancy covariates, with effects more pronounced in females compared to males. These findings support the evidence linking different patterns of fetal growth and markers of adult cardiovascular disease, including our finding of such relationships with adult blood pressure in the same cohort [30].

In our study, the trajectories that associated with long-term adiposity showed discordance in growth between early and later pregnancy. In particular, 38% of the offspring that experienced average or greater growth of abdominal circumference during the first half of gestation (with diminishing late growth), associated with 1 kg/m^2^ and 1.45 kg/m^2^, respectively, lower adult BMI. Greater-than-average abdominal circumference during the first half of gestation (with diminishing late growth) also associated with a 3.1 cm lower waist circumference in adulthood. Whereas there was no association between the consistently low AC trajectory and adult BMI, lower fetal femur length in early pregnancy followed by accelerated growth late in pregnancy associated with 2.58 kg/m^2^ higher adult BMI. Similarly lower head circumference in early pregnancy (which increased in late pregnancy) in females was associated with higher adult adiposity.

Data from the Generation R Study on 1184 children with first-trimester fetal crown to rump length measurements showed that impaired first-trimester fetal growth was associated with adverse cardiovascular risk in school age children and reported 0.3% lower total fat mass for every one standard deviation higher first-trimester fetal length [41]. In another Generation R study performed on a cohort of 481 healthy children, there was a tendency towards inverse association of estimated fetal weight in the second trimester with preperitoneal fat at 2 years of age [42]. Using femur length and head circumference, we similarly observed higher BMI in some fetuses with less than average size that is already apparent by middle of second trimester. Early fetal growth serves as a critical period in relation to adult adiposity. Our study adds further to evidence for this concept, by showing that not only does restricted early growth associate with future adiposity but sufficient growth in early pregnancy is associated with a protective effect on adiposity later in life. Our data shows that a very-low-to-rising FL trajectory associated with higher adult BMI which is consistent with studies such as the Danish Fetal Origin Cohort 1988 (DaFO88) and Generation R [43, 44]. The DaFO88 study showed that an upward change in growth trajectory, defined using bi-parietal diameter difference at 20-weeks gestation and birth, was associated with increased BMI at 20 years [43]. The Generation R Study found that accelerated third-trimester fetal growth was associated with an increased risk of overweight in preschool children [44]. Our study builds on this evidence by showing the association between accelerated fetal growth and increased risk of adiposity at 27 years.

The relationship between postnatal measures including birthweight and adiposity during adolescence and adulthood has been extensively examined [45–50]. Systematic reviews and meta-analyses have reported a positive association between high birthweight and increased risk of adiposity from childhood to adulthood [13–16, 47, 50]. Although our birthweight findings are reflective of this, the use of serial ultrasound measures in this study shows that birthweight does not accurately represent different trajectories of early-mid fetal growth. We have previously shown that offspring with similar birthweight can have substantially different fetal growth patterns, and fetuses with similar growth at 15 weeks gestation can have a significantly different birthweight [30]. Such differences may play an important role in determining adiposity and cardiometabolic health in adolescence and adulthood. The associations between trajectory groups and adult outcomes in this study were independent of maternal factors during pregnancy, although in some instances the analysis may not have had sufficient power to achieve statistical significance based on the global P-value. Several maternal factors such as uncomplicated hypertension during pregnancy, maternal alcohol drinking, and maternal BMI at 16 weeks were significantly associated with adult adiposity and hs-CRP. Our findings thus contribute to the existing evidence that the effect of early intrauterine environment on fetal growth could be an important determinant of adiposity in later life.

Evidence for the key role of environmental influences on subsequent childhood and adult adiposity comes from several experimental animal studies and birth cohort studies, as well as a few human trials [51–55]. These studies have shown the influence of maternal BMI and smoking, pre-eclampsia, and gestational weight gain, on childhood obesity. Different mechanisms proposed include the role of fetal adipocytes, leptin, epigenetic modifications and exposure to glucocorticoids [11, 56]. Maternal stress, anxiety, and depression during pregnancy can lead to increased fetal exposure to glucocorticoids and activation of the hypothalamic-pituitary adrenal axis which plays an important role in fetal development and subsequent risk of adiposity in later life [57–59].

Our findings are the first to show an inverse relationship between measures of in utero fetal growth and adult hs-CRP, a biomarker of chronic low-grade systemic inflammation. While restricted fetal abdominal and femur growth both associated with higher hs-CRP in adulthood, greater-than-average fetal abdominal and head growth associated with lower adult hs-CRP, independently of adult BMI and maternal factors. These data add to the existing literature that show an inverse association between birthweight and hs-CRP in children and adults [60]. Chronic low-grade inflammation is the hallmark of obesity and increased levels of inflammatory mediators have been found in individuals with obesity [61]. In utero undernutrition can cause permanent injury to fat depots, liver, and muscle tissue and lead to a state of low-grade inflammation postnatally [60]. Recent data from the Raine Study have also shown a strong association between hs-CRP and BMI in the offspring from the age of 14 years to 22 years [62]. Our findings showed some differences between associations with adiposity and hs-CRP. The associations with hs-CRP tended to be with the extreme largest and smallest groups that maintained their relative size throughout pregnancy. Additionally, the effect was independent of maternal factors, and no female preponderance of effect was observed.

Obesity is a complex phenomenon, involving an array of genetic and environmental factors. While the postnatal factors contributing to adiposity have been extensively studied, there is limited literature investigating the antenatal origins of adult obesity using serial ultrasound measures. Previous antenatal studies have focussed on childhood obesity and very few have used serial fetal anthropometric measurements to examine fetal growth [28, 63]. In our study, the associations between growth trajectories, anthropometry, and hs-CRP were adjusted for age and sex, with associations persisting after adjusting for postnatal lifestyle factors. We also found significant sex differences between trajectories and the outcome measures: BMI and waist circumference associations were evident in females, whilst an association with hs-CRP was only in males.

Our limited sample size and the number of trajectory groups did not allow us to explore the mechanisms underlying maternal or adult lifestyle factors. Nevertheless, the pregnancy and birth characteristics of the different trajectory groups for all three fetal anthropometric markers has been described previously [30]. In particular, mean maternal BMI at 16 weeks gestation was 23.1 kg/m^2^ and 23.6 kg/m^2^ for Groups-4 and 6 respectively for abdominal circumference, compared to 24.3 kg/m^2^ for the reference Group-3. None of the mothers of participants in Group-5 for the head circumference trajectory reported complicated hypertension during pregnancy, compared to 2.2% in the reference Group-4.

Strengths of this study include the use of serial fetal growth ultrasound measurements taken during pregnancy to assess fetal growth and examine the association with measures of adult adiposity and inflammation. The Raine Study cohort comprises of a well-characterized group of individuals with carefully documented antenatal and postnatal information. Despite availability of approximately 50% of the cohort, it represents the contemporary Western Australian population both at the recruitment and during each follow-up which allows generalization of the findings to similar populations [31, 37, 64]. The robustness of linear mixed modeling along with bootstrapping allowed us to investigate the relationship between fetal growth and adiposity markers [39, 40]. The breadth of data available in the Raine Study enabled us to explore relationships independent of adult and maternal factors influencing adiposity. A limitation of this study was that serial ultrasound measures were conducted not prior to 15 weeks of gestation, consequently we don’t have data on the actual first-trimester growth patterns. However, assessment of fetal anthropometric markers at 15 weeks is likely to represent first-trimester growth and along with subsequent measurements, it provided substantial information to make a reliable estimation of fetal growth throughout gestation. Another limitation was our inability to explore sex-trajectory interactions using multiple comparisons due to small sample and sizable number of trajectory groups. There was no adjustment for sibling rank due to non-availability of information. However, mother’s parity was accounted for while constructing the trajectories. Lastly, it is important to understand that this is an observational study and causality inferences need a cautious approach.

Our study has shown that growth patterns established in utero associate with adult markers of anthropometry and inflammation which are related to the risk of future cardiovascular disease. These data together with our previous results that showed restricted fetal growth associated with higher adult blood pressure, add to our understanding of the concept of the developmental origins of health and disease and provide new evidence of antenatal determinants of adiposity in adult life. To the best of our knowledge, this is the first study to have shown a relationship between fetal growth as early as 15 weeks and adult adiposity using serial ultrasound-based trajectory modeling in a well-structured cohort. The results reinforce evidence for a role of fetal programming and early life environmental influences on adult adiposity and low-grade inflammation. Preventive health care interventions targeting factors affecting maternal and fetal well-being including pregnancy diseases such as gestational diabetes and pre-eclampsia, cigarette smoking, eating habits, and hormonal imbalances could potentially play a vital role in determining future cardiometabolic risk. Future research is needed to establish mechanisms driving the unique in utero growth patterns and the definitive pathways leading to the enhanced risk of adiposity and inflammatory-related disorders in later life.

## Supplementary information

Supplementary Tables and Figures

## Data Availability

The datasets generated during and/or analyzed during the current study are not available. The Raine Study is committed to a high level of confidentiality of the data in line with the informed consent provided by participants. Requests for data should be directed to the Raine Study Executive.
