# Supplementary material for: Relationships between intrauterine fetal growth trajectories and markers of adiposity and inflammation in young adults
Source: Int J Obes (Lond). 2022 Aug 17;46(10):1925–35. doi: 10.1038/s41366-022-01203-2 (PMC9492546; doi:10.1038/s41366-022-01203-2)
Supplement: Supplementary file 1 — Supplementary Tables and Figures [file 41366_2022_1203_MOESM1_ESM.docx]

**Supplementary Table-1: Abdominal circumference (AC) growth trajectories *in-utero* and their association with BMI, WC & hs-CRP (Model 1)**

| **BMI (Model 1)*** | **Regression coefficient** | **95 % CI** | | **P-value** |
| --- | --- | --- | --- | --- |
| Trajectory Groups (*Ref- Group-3*) |  |  | | **0.011 (global)** |
| Group-1 | -0.06 | -1.67 | 1.55 | 0.940 |
| Group-2 | -0.31 | -1.56 | 0.94 | 0.627 |
| **Group-4** | **-1.45** | **-2.43** | **-0.46** | **0.004** |
| Group-5 | 0.26 | -0.87 | 1.38 | 0.654 |
| **Group-6** | **-1.01** | **-1.96** | **-0.05** | **0.038** |
| Group-7 | 0.20 | -1.36 | 1.76 | 0.802 |
| Follow up Year (*Ref- Y20*) |  |  | | < 0.001 (global) |
| Y22 | 0.71 | 0.45 | 0.96 | < 0.001 |
| Y27 | 1.43 | 1.06 | 1.80 | < 0.001 |
| Sex- Males | -0.17 | -0.94 | 0.60 | 0.661 |
| Contraceptive use in females | -0.40 | -0.88 | 0.07 | 0.093 |
| Ethnicity | 2.03 | 1.03 | 1.80 | < 0.001 |
| Socio-economic status | -1.05 | -1.39 | -0.71 | < 0.001 |
| Physical activity (MET mins/week) | -0.22 | -0.42 | -0.03 | 0.027 |
| *Intercept* | 23.36 | 22.03 | 24.69 | < 0.001 |
| **WC (Model 1)*** | **Regression coefficient** | **95 % CI** | | **P-value** |
| Trajectory Groups (*Ref- Group-3*) |  |  | | **0.033 (global)** |
| Group-1 | 0.44 | -3.76 | 4.63 | 0.838 |
| Group-2 | -1.06 | -4.02 | 1.90 | 0.482 |
| **Group-4** | **-3.10** | **-5.48** | **-0.72** | **0.011** |
| Group-5 | 0.78 | -1.85 | 3.40 | 0.561 |
| Group-6 | -1.86 | -4.13 | 0.40 | 0.107 |
| Group-7 | 0.16 | -3.78 | 4.10 | 0.938 |
| Follow up Year (*Ref- Y20*) |  |  | | < 0.001 (global) |
| Y22 | 3.31 | 2.47 | 4.14 | < 0.001 |
| Y27 | 4.67 | 3.51 | 5.84 | < 0.001 |
| Sex- Males | 5.27 | 3.34 | 7.20 | < 0.001 |
| Contraceptive use in females | -1.74 | -3.22 | -0.27 | 0.021 |
| Ethnicity | 6.05 | 3.89 | 8.20 | < 0.001 |
| Socio-economic status | -2.70 | -3.47 | -1.93 | < 0.001 |
| Physical activity (MET mins/week) | -0.75 | -1.34 | -0.15 | 0.013 |
| *Intercept* | 73.49 | 70.55 | 76.42 | < 0.001 |
| **hs-CRP (exponentiated) (Model 1)**** | **Regression coefficient** | **95 % CI** | | **P-value** |
| Trajectory Groups (*Ref- Group-3*) |  |  | | **0.001 (global)** |
| **Group-1** | **1.40** | **1.07** | **1.83** | **0.013** |
| Group-2 | 1.07 | 0.87 | 1.32 | 0.526 |
| Group-4 | 1.05 | 0.88 | 1.25 | 0.62 |
| Group-5 | 0.99 | 0.81 | 1.22 | 0.948 |
| Group-6 | 0.98 | 0.80 | 1.20 | 0.839 |
| **Group-7** | **0.70** | **0.56** | **0.88** | **0.002** |
| Follow up Year (*Ref- Y20*) |  |  | | < 0.001 (global) |
| Y22 | 0.83 | 0.71 | 0.97 | 0.017 |
| Y27 | 0.73 | 0.62 | 0.85 | < 0.001 |
| Sex- Males | 0.81 | 0.69 | 0.94 | 0.007 |
| Contraceptive use in females | 2.01 | 1.66 | 2.43 | < 0.001 |
| Ethnicity | 1.29 | 1.04 | 1.59 | 0.020 |
| Socio-economic status | 0.96 | 0.90 | 1.03 | 0.307 |
| BMI centered at 25 kg/m^2^ | 1.11 | 1.10 | 1.13 | < 0.001 |
| Physical activity (MET mins/week) | 0.91 | 0.84 | 0.97 | 0.006 |
| *Intercept* | 0.88 | 0.68 | 1.15 | 0.359 |

Linear mixed modelling results displayed; ^#^Trajectories based on 1333 participants; Reference group is Group-3 (Average-stable) with a membership of 24.9% (n=169); *Model results for BMI & WC based on 1242 observations representing 606 participants who attended one or more follow up. **Model results for hs-CRP based on 1139 observations representing 574 participants who attended one or more follow up.

**Supplementary Table-2: Abdominal circumference (AC) growth trajectories *in-utero* and their association with BMI, WC & hs-CRP (Model 2)**

| **BMI (Model 2)*** | **Regression coefficient** | **95 % CI** | | **P-value** |
| --- | --- | --- | --- | --- |
| Trajectory Groups (*Ref- Group-3*) |  |  | | **0.044 (global)** |
| Group-1 | 0.86 | -0.76 | 2.49 | 0.297 |
| Group-2 | 0.21 | -0.94 | 1.37 | 0.719 |
| **Group-4** | **-1.03** | **-1.84** | **-0.23** | **0.012** |
| Group-5 | -0.04 | -1.14 | 1.06 | 0.946 |
| Group-6 | -0.75 | -1.67 | 0.17 | 0.111 |
| Group-7 | -0.47 | -1.91 | 0.97 | 0.524 |
| Follow up Year (*Ref- Y20*) |  |  | | < 0.001 (global) |
| Y22 | 0.70 | 0.44 | 0.96 | < 0.001 |
| Y27 | 1.44 | 1.03 | 1.86 | < 0.001 |
| Sex- Males | -0.19 | -0.91 | 0.53 | 0.610 |
| Contraceptive use in females | -0.40 | -0.85 | 0.06 | 0.088 |
| Ethnicity | 1.16 | 0.16 | 2.16 | 0.023 |
| Socio-economic status | -0.58 | -0.93 | -0.24 | 0.001 |
| Physical activity (MET mins/week) | -0.23 | -0.44 | -0.02 | 0.030 |
| Uncomplicated Hypertension | 1.18 | 0.28 | 2.08 | 0.010 |
| Maternal BMI at 16-weeks | 1.96 | 1.53 | 2.38 | < 0.001 |
| Maternal Alcohol drinking | -0.92 | -1.59 | -0.26 | 0.006 |
| *Intercept* | 24.30 | 23.06 | 25.53 | < 0.001 |
| **WC (Model 2)*** | **Regression coefficient** | **95 % CI** | | **P-value** |
| Trajectory Groups (*Ref- Group-3*) |  |  | | 0.138 (global) |
| Group-1 | 2.56 | -1.76 | 6.87 | 0.245 |
| Group-2 | 0.24 | -2.56 | 3.04 | 0.867 |
| **Group-4** | **-2.20** | **-4.16** | **-0.24** | **0.028** |
| Group-5 | 0.20 | -2.42 | 2.83 | 0.879 |
| Group-6 | -1.19 | -3.35 | 0.97 | 0.279 |
| Group-7 | -1.38 | -4.77 | 2.01 | 0.425 |
| Follow up Year (*Ref- Y20*) |  |  | | < 0.001 (global) |
| Y22 | 3.29 | 2.47 | 4.11 | < 0.001 |
| Y27 | 4.67 | 3.49 | 5.85 | < 0.001 |
| Sex- Males | 5.27 | 3.51 | 7.04 | < 0.001 |
| Contraceptive use in females | -1.70 | -3.17 | -0.22 | 0.024 |
| Ethnicity | 4.30 | 2.16 | 6.44 | < 0.001 |
| Socio-economic status | -1.60 | -2.39 | -0.81 | < 0.001 |
| Physical activity (MET mins/week) | -0.77 | -1.38 | -0.16 | 0.013 |
| Uncomplicated Hypertension | 2.27 | 0.19 | 4.36 | 0.032 |
| Maternal BMI at 16-weeks | 4.38 | 3.38 | 5.39 | < 0.001 |
| Maternal Alcohol drinking | -2.79 | -4.43 | -1.16 | 0.001 |
| *Intercept* | 75.84 | 73.07 | 78.61 | < 0.001 |
| **hs-CRP (exponentiated) (Model 2)**** | **Regression coefficient** | **95 % CI** | | **P-value** |
| Trajectory Groups (*Ref- Group-3*) |  |  | | **0.001 (global)** |
| **Group-1** | **1.48** | **1.13** | **1.93** | **0.005** |
| Group-2 | 1.07 | 0.87 | 1.32 | 0.525 |
| Group-4 | 1.06 | 0.88 | 1.28 | 0.529 |
| Group-5 | 0.98 | 0.79 | 1.22 | 0.885 |
| Group-6 | 0.97 | 0.80 | 1.18 | 0.796 |
| **Group-7** | **0.70** | **0.55** | **0.88** | **0.002** |
| Follow up Year (*Ref- Y20*) |  |  | | < 0.001 (global) |
| Y22 | 0.82 | 0.71 | 0.97 | 0.017 |
| Y27 | 0.72 | 0.62 | 0.85 | < 0.001 |
| Sex- Males | 0.82 | 0.69 | 0.94 | 0.007 |
| Contraceptive use in females | 2.01 | 1.66 | 2.43 | < 0.001 |
| Ethnicity | 1.29 | 1.04 | 1.59 | 0.020 |
| Socio-economic status | 0.96 | 0.90 | 1.03 | 0.307 |
| BMI centered at 25 kg/m^2^ | 1.11 | 1.10 | 1.13 | < 0.001 |
| Physical activity (MET mins/week) | 0.90 | 0.84 | 0.97 | 0.006 |
| Maternal BMI at 16-weeks | 1.03 | 1.01 | 1.09 | 0.036 |
| *Intercept* | 0.88 | 0.68 | 1.15 | 0.359 |

Linear mixed modelling results displayed; ^#^Trajectories based on 1333 participants; Reference group is Group-3 (Average-stable) with a membership of 24.9% (n=169); *Model results for BMI & WC based on 1223 observations representing 596 participants who attended one or more follow up. **Model results for hs-CRP based on 1126 observations representing 566 participants who attended one or more follow up.

**Supplementary Table-3: BMI categories across Abdominal Circumference (AC) trajectory groups at 20, 22 and 27-years**

| Follow up | **Trajectories*** | | Group-1  Low-falling  (n=36) | Group-2  Low-stable  (n=89) | **Group-3 Average-stable (Reference^#^)**  **(n=169)** | | Group-4  Average-falling  (n=139) | Group-5  Average-rising  (n=84) | Group-6  High-falling  (n=124) | Group-7  High-rising  (n=38) |
| --- | --- | --- | --- | --- | --- | --- | --- | --- | --- | --- |
|  | | | | | | | | | | |
| 20-years | **# of adults (N)** | **24** | | **63** | **117** | **101** | | **56** | **99** | **25** |
|  | <25 (kg/m^2^) | 15  (62.5) | | 42  (66.7) | 75  (64.1) | 73  (72.3) | | 34  (60.7) | 66  (66.7) | 16  (64.0) |
|  | Overweight | 4  (16.7) | | 10  (15.9) | 29  (24.8) | 18  (17.8) | | 12  (21.4) | 23  (23.2) | 3  (12.0) |
|  | Obese | 5  (20.8) | | 11  (17.5) | 13  (11.1) | 10  (9.9) | | 10  (17.9) | 10  (10.1) | 6  (24.0) |
| 22-years | **# of adults (N)** | **21** | | **50** | **108** | **92** | | **51** | **72** | **27** |
|  | <25 (kg/m^2^) | 12  (57.1) | | 31  (62.0) | 64  (59.3) | 62  (67.4) | | 24  (47.0) | 47  (65.3) | 17  (63.0) |
|  | Overweight | 1  (4.8) | | 12  (24.0) | 24  (22.2) | 20  (21.7) | | 21  (41.2) | 16  (22.2) | 4  (14.8) |
|  | Obese | 8  (38.1) | | 7  (14.0) | 20  (18.5) | 10  (10.9) | | 6  (11.8) | 9  (12.5) | 6  (22.2) |
| 27-years | **# of adults (N)** | **21** | | **57** | **112** | **85** | | **54** | **77** | **31** |
|  | <25 (kg/m^2^) | 11  (52.4) | | 28  (49.1) | 55  (49.1) | 56  (65.8) | | 27  (50.0) | 53  (68.8) | 17  (54.8) |
|  | Overweight | 6  (28.6) | | 20  (35.1) | 32  (28.6) | 23  (27.1) | | 14  (25.9) | 16  (20.8) | 8  (25.8) |
|  | Obese | 4  (19.0) | | 9  (15.8) | 25  (22.3) | 6  (7.1) | | 13  (24.1) | 8  (10.4) | 6  (19.4) |

Values represent frequencies with column percentages in parentheses *Trajectories based on 1333 participants, 679 of them analysed for BMI outcome; ^#^Reference group representing average growth; *BMI Categories: <25, Overweight (≥25 and <30), Obese (≥30) [in kg/m^2^]

**Supplementary Table-4: Femur length (FL) growth trajectories *in-utero***

**and their association with BMI, WC & hs-CRP (Model 1)**

| **BMI (Model 1)*** | **Regression coefficient** | **95 % CI** | | **P-value** |
| --- | --- | --- | --- | --- |
| Trajectory Groups (*Ref- Group-4*) |  |  | | **0.028 (global)** |
| Group-1 | 0.50 | -1.33 | 2.33 | 0.593 |
| **Group-2** | **2.58** | **0.98** | **4.18** | **0.002** |
| Group-3 | 0.45 | -0.27 | 1.16 | 0.220 |
| Group-5 | 0.35 | -0.66 | 1.36 | 0.499 |
| Follow up Year (*Ref- Y20*) |  |  | | < 0.001 (global) |
| Y22 | 0.70 | 0.45 | 0.96 | < 0.001 |
| Y27 | 1.43 | 1.06 | 1.80 | < 0.001 |
| Sex- Males | -0.21 | -1.00 | 0.58 | 0.598 |
| Contraceptive use in females | -0.41 | -0.88 | 0.07 | 0.091 |
| Ethnicity | 2.13 | 1.12 | 3.14 | < 0.001 |
| Socio-economic status | -1.07 | -1.41 | -0.74 | < 0.001 |
| Physical Activity (MET mins/week) | -0.22 | -0.42 | -0.03 | 0.026 |
| *Intercept* | 22.41 | 21.32 | 23.50 | < 0.001 |
| **WC (Model 1)*** | **Regression coefficient** | **95 % CI** | | **P-value** |
| Trajectory Groups (*Ref- Group-4*) |  |  | | 0.055 (global) |
| Group-1 | -1.39 | -5.28 | 2.51 | 0.486 |
| **Group-2** | **5.22** | **1.28** | **9.17** | **0.009** |
| Group-3 | 0.37 | -1.37 | 2.12 | 0.674 |
| Group-5 | 2.04 | -0.46 | 4.54 | 0.11 |
| Follow up Year (*Ref- Y20*) |  |  | | < 0.001 (global) |
| Y22 | 3.32 | 2.48 | 4.15 | < 0.001 |
| Y27 | 4.68 | 3.52 | 5.84 | < 0.001 |
| Sex- Males | 5.24 | 3.29 | 7.18 | < 0.001 |
| Contraceptive use in females | -1.76 | -3.24 | -0.28 | 0.020 |
| Ethnicity | 6.16 | 4.01 | 8.31 | < 0.001 |
| Socio-economic status | -2.77 | -3.53 | -2.02 | < 0.001 |
| Physical activity (MET mins/week) | -0.74 | -1.33 | -0.15 | 0.014 |
| *Intercept* | 71.71 | 69.30 | 74.13 | < 0.001 |
| **hs-CRP (exponentiated) (Model 1)**** | **Regression coefficient** | **95 % CI** | | **P-value** |
| Trajectory Groups (*Ref- Group-4*) |  |  | | **< 0.001 (global)** |
| Group-1 | 1.13 | 0.88 | 1.46 | 0.326 |
| **Group-2** | **1.37** | **1.09** | **1.73** | **0.008** |
| **Group-3** | **1.37** | **1.19** | **1.58** | **< 0.001** |
| Group-5 | 1.12 | 0.92 | 1.35 | 0.255 |
| Follow up Year (*Ref- Y20*) |  |  | | < 0.001 (global) |
| Y22 | 0.83 | 0.71 | 0.97 | 0.016 |
| Y27 | 0.75 | 0.65 | 0.86 | < 0.001 |
| Sex- Males | 0.80 | 0.69 | 0.94 | 0.005 |
| Contraceptive use in females | 1.97 | 1.63 | 2.39 | < 0.001 |
| Ethnicity | 1.28 | 1.03 | 1.59 | 0.025 |
| Socio-economic status | 0.96 | 0.90 | 1.03 | 0.238 |
| BMI centered at 25 kg/m^2^ | 1.11 | 1.10 | 1.13 | < 0.001 |
| Physical activity (MET mins/week) | 0.91 | 0.85 | 0.97 | 0.007 |
| *Intercept* | 0.78 | 0.61 | 1.01 | 0.058 |

Linear mixed modelling results displayed. ^#^Trajectories based on 1333 participants; Reference group: Group-4 (Average-stable) with a membership of 42.4% (n=288); *Model results for BMI & WC based on 1242 observations representing 606 participants who attended one or more follow up. **Model results for hs-CRP based on 1139 observations representing 574 participants who attended one or more follow up.

**Supplementary Table-5: Femur length (FL) growth trajectories *in-utero***

**and their association with BMI, WC & hs-CRP (Model 2)**

| **BMI (Model 2)*** | **Regression coefficient** | **95 % CI** | | **P-value** |
| --- | --- | --- | --- | --- |
| Trajectory Groups (*Ref- Group-4*) |  |  | | 0.159 (global) |
| Group-1 | 0.39 | -1.16 | 1.93 | 0.622 |
| **Group-2** | **1.77** | **0.20** | **3.35** | **0.027** |
| Group-3 | 0.50 | -0.19 | 1.19 | 0.156 |
| Group-5 | 0.05 | -0.89 | 1.00 | 0.912 |
| Follow up Year (*Ref- Y20*) |  |  | | < 0.001 (global) |
| Y22 | 0.70 | 0.44 | 0.96 | < 0.001 |
| Y27 | 1.44 | 1.03 | 1.85 | < 0.001 |
| Sex- Males | -0.20 | -0.93 | 0.53 | 0.583 |
| Contraceptive use in females | -0.40 | -0.86 | 0.05 | 0.082 |
| Ethnicity | 1.26 | 0.25 | 2.27 | 0.014 |
| Socio-economic status | -0.61 | -0.95 | -0.27 | < 0.001 |
| Physical Activity (MET mins/week) | -0.23 | -0.44 | -0.02 | 0.030 |
| Uncomplicated Hypertension | 1.09 | 0.21 | 1.97 | 0.015 |
| Maternal BMI at 16-weeks | 1.94 | 1.52 | 2.36 | < 0.001 |
| Maternal Alcohol drinking | -0.90 | -1.56 | -0.24 | 0.008 |
| *Intercept* | 23.59 | 22.51 | 24.67 | < 0.001 |
| **WC (Model 2)*** | **Regression coefficient** | **95 % CI** | | **P-value** |
| Trajectory Groups (*Ref- Group-4*) |  |  | | 0.296 (global) |
| Group-1 | -1.62 | -5.06 | 1.83 | 0.357 |
| Group-2 | 3.42 | -0.30 | 7.14 | 0.071 |
| Group-3 | 0.47 | -1.14 | 2.07 | 0.569 |
| Group-5 | 1.29 | -1.07 | 3.64 | 0.284 |
| Follow up Year (*Ref- Y20*) |  |  | | < 0.001 (global) |
| Y22 | 3.30 | 2.48 | 4.12 | < 0.001 |
| Y27 | 4.68 | 3.50 | 5.86 | < 0.001 |
| Sex- Males | 5.27 | 3.47 | 7.08 | < 0.001 |
| Contraceptive use in females | -1.73 | -3.21 | -0.25 | 0.022 |
| Ethnicity | 4.49 | 2.33 | 6.64 | < 0.001 |
| Socio-economic status | -1.67 | -2.45 | -0.90 | < 0.001 |
| Physical activity (MET mins/week) | -0.76 | -1.36 | -0.15 | 0.015 |
| Uncomplicated Hypertension | 2.23 | 0.23 | 4.22 | 0.029 |
| Maternal BMI at 16-weeks | 4.30 | 3.30 | 5.29 | < 0.001 |
| Maternal Alcohol drinking | -2.79 | -4.40 | -1.17 | 0.001 |
| *Intercept* | 74.65 | 72.15 | 77.14 | < 0.001 |
| **hs-CRP (exponentiated) (Model 2)**** | **Regression coefficient** | **95 % CI** | | **P-value** |
| Trajectory Groups (*Ref- Group-4*) |  |  | | **< 0.001 (global)** |
| Group-1 | 1.13 | 0.86 | 1.48 | 0.373 |
| **Group-2** | **1.38** | **1.09** | **1.74** | **0.007** |
| **Group-3** | **1.39** | **1.21** | **1.61** | **< 0.001** |
| Group-5 | 1.12 | 0.94 | 1.35 | 0.211 |
| Follow up Year (*Ref- Y20*) |  |  | | < 0.001 (global) |
| Y22 | 0.82 | 0.70 | 0.95 | 0.011 |
| Y27 | 0.72 | 0.62 | 0.85 | < 0.001 |
| Sex- Males | 0.82 | 0.69 | 0.96 | 0.017 |
| Contraceptive use in females | 1.97 | 1.61 | 2.41 | < 0.001 |
| Ethnicity | 1.30 | 1.05 | 1.61 | 0.017 |
| Socio-economic status | 0.96 | 0.90 | 1.02 | 0.150 |
| BMI centered at 25 kg/m^2^ | 1.11 | 1.10 | 1.13 | < 0.001 |
| Physical activity (MET mins/week) | 0.90 | 0.84 | 0.97 | 0.005 |
| Maternal BMI at 16-weeks | 1.07 | 1.01 | 1.16 | 0.021 |
| *Intercept* | 0.77 | 0.60 | 0.99 | 0.040 |

Linear mixed modelling results displayed. ^#^Trajectories based on 1333 participants; Reference group: Group-4 (Average-stable) with a membership of 42.4% (n=288); *Model results for BMI & WC based on 1223 observations representing 596 participants who attended one or more follow up. **Model results for hs-CRP based on 1126 observations representing 566 participants who attended one or more follow up.

**Supplementary Table-6: BMI categories across Femur Length (FL) trajectory groups**

**at 20, 22 and 27-years**

| Follow up | **Trajectories*** | Group-1  Low-falling  (n=40) | Group-2  Very-low rising  (n=52) | Group-3  Low-stable  (n=215) | **Group-4**  **Average-stable**  **(*Reference)**  **(n=288)** | Group-5  High-stable  (n=84) |
| --- | --- | --- | --- | --- | --- | --- |
| 20-years | **# of adults (N)** | **25** | **32** | **155** | **210** | **63** |
|  | <25 (kg/m^2^) | 21  (84.0) | 20  (62.5) | 94  (60.6) | 148  (70.5) | 38  (60.3) |
|  | Overweight | 1  (4.0) | 7  (21.9) | 37  (23.9) | 40  (19.0) | 14  (22.2) |
|  | Obese | 3  (12.0) | 5  (15.6) | 24  (15.5) | 22  (10.5) | 11  (17.5) |
| 22-years | **# of adults (N)** | **28** | **32** | **133** | **172** | **56** |
|  | <25 (kg/m^2^) | 18  (64.2) | 19  (59.4) | 76  (57.1) | 107  (62.2) | 37  (66.1) |
|  | Overweight | 5  (17.8) | 7  (21.8) | 35  (26.3) | 41  (23.8) | 10  (17.8) |
|  | Obese | 5  (17.8) | 6  (18.8) | 22  (16.6) | 24  (14.0) | 9  (16.1) |
| 27-years | **# of adults (N)** | **29** | **36** | **132** | **182** | **58** |
|  | <25 (kg/m^2^) | 13  (44.8) | 12  (33.3) | 74  (56.1) | 109  (59.9) | 39  (67.2) |
|  | Overweight | 13  (44.8) | 16  (44.4) | 30  (22.7) | 50  (27.5) | 10  (17.2) |
|  | Obese | 3  (10.4) | 8  (22.2) | 28  (21.2) | 23  (12.6) | 9  (15.5) |

Values represent frequencies with column percentages in parentheses *Trajectories based on 1333 participants, 679 of them analysed for BMI outcome; ^#^Reference group represents average growth; *BMI Categories: <25, Overweight (≥25 and <30), Obese (≥30) [in kg/m^2^]

**Supplementary Table-7: Femur Length (FL) growth trajectories *in-utero***

**and their association with hs-CRP in both sexes**

| ^#^Trajectories (Group membership) | Group-1  Low-falling  8.0%  (n=26) | Group-2  Very low-rising  7.7%  (n=25) | Group-3  Low-stable  31.7%  (n=103) | Group-5  High-stable  11.7%  (n=38) | Global  P-value |
| --- | --- | --- | --- | --- | --- |
|  | **Males: hs-CRP** | | | |  |
| Model 1 | 1.14 | 1.74*** | 1.10 | 1.09 | 0.035 |
| Model 2 | 1.14 | 1.74** | 1.13 | 1.10 | 0.042 |
|  | | | | | |
| ^#^Trajectories (Group membership) | Group-1  Low-falling  3.8%  (n=12) | Group-2  Very low-rising  8.2%  (n=26) | Group-3  Low-stable  32.0%  (n=101) | Group-5  High-stable  13.3%  (n=42) | Global  P-value |
|  | **Females: hs-CRP** | | | |  |
| Model 1 | 1.07 | 1.12 | 1.70*** | 1.14 | <0.001 |
| Model 2 | 1.08 | 1.13 | 1.70*** | 1.15 | <0.001 |

Linear mixed modelling results displayed (exponentiated β coefficients).

Significant P-values: **<0.01, ***<0.001.

^#^Trajectories based on 1333 participants; Reference group is Group-4 (Average-stable) with a membership of 40.9% (n=133) for males and 42.7% (n=135) for females.

Model 1 covariates- age, ethnicity, SES, BMI & physical activity; Model 2 covariates- Model 1 plus maternal BMI at 16-weeks.

**Supplementary Table-8: Head circumference (HC) growth trajectories *in-utero* and their association with BMI, WC & hs-CRP (Model 1)**

| **BMI (Model 1)*** | **Regression coefficient** | **95 % CI** | | **P-value** |
| --- | --- | --- | --- | --- |
| Trajectory Groups (*Ref- Group-4*) |  |  | | 0.488 (global) |
| Group-1 | -0.55 | -1.97 | 0.88 | 0.451 |
| Group-2 | -0.10 | -0.88 | 0.68 | 0.799 |
| Group-3 | 0.57 | -0.72 | 1.86 | 0.388 |
| Group-5 | -0.61 | -1.55 | 0.33 | 0.207 |
| Follow up Year (*Ref- Y20*) |  |  | | < 0.001 (global) |
| Y22 | 0.71 | 0.45 | 0.96 | < 0.001 |
| Y27 | 1.43 | 1.06 | 1.81 | < 0.001 |
| Sex- Males | -0.19 | -0.96 | 0.58 | 0.631 |
| Contraceptive use in females | -0.40 | -0.87 | 0.07 | 0.095 |
| Ethnicity | 2.12 | 1.09 | 3.14 | < 0.001 |
| Socio-economic status | -1.06 | -1.39 | -0.73 | < 0.001 |
| Physical activity (MET mins/week) | -0.22 | -0.42 | -0.03 | 0.026 |
| *Intercept* | 22.92 | 21.77 | 24.06 | < 0.001 |
| **WC (Model 1)*** | **Regression coefficient** | **95 % CI** | | **P-value** |
| Trajectory Groups (*Ref- Group-4*) |  |  | | 0.714 (global) |
| Group-1 | -0.95 | -4.32 | 2.42 | 0.580 |
| Group-2 | -0.76 | -2.63 | 1.11 | 0.427 |
| Group-3 | 1.15 | -1.93 | 4.24 | 0.464 |
| Group-5 | -0.89 | -3.30 | 1.53 | 0.473 |
| Follow up Year (*Ref- Y20*) |  |  | | < 0.001 (global) |
| Y22 | 3.31 | 2.48 | 4.14 | < 0.001 |
| Y27 | 4.68 | 3.52 | 5.84 | < 0.001 |
| Sex- Males | 5.15 | 3.21 | 7.08 | < 0.001 |
| Contraceptive use in females | -1.75 | -3.22 | -0.27 | 0.021 |
| Ethnicity | 6.25 | 4.09 | 8.41 | < 0.001 |
| Socio-economic status | -2.73 | -3.49 | -1.98 | < 0.001 |
| Physical activity (MET mins/week) | -0.75 | -1.34 | -0.16 | 0.013 |
| *Intercept* | 72.70 | 70.14 | 75.25 | < 0.001 |
| **hs-CRP (exponentiated) (Model 1)**** | **Regression coefficient** | **95 % CI** | | **P-value** |
| Trajectory Groups (*Ref- Group-4*) |  |  | | **0.020 (global)** |
| Group-1 | 1.16 | 0.91 | 1.49 | 0.222 |
| Group-2 | 1.05 | 0.91 | 1.21 | 0.519 |
| Group-3 | 1.02 | 0.82 | 1.26 | 0.867 |
| **Group-5** | **0.80** | **0.67** | **0.95** | **0.011** |
| Follow up Year (*Ref- Y20*) |  |  | | < 0.001 (global) |
| Y22 | 0.83 | 0.71 | 0.97 | 0.017 |
| Y27 | 0.73 | 0.63 | 0.85 | < 0.001 |
| Sex- Males | 0.82 | 0.70 | 0.96 | 0.012 |
| Contraceptive use in females | 2.01 | 1.66 | 2.43 | < 0.001 |
| Ethnicity | 1.31 | 1.06 | 1.62 | 0.013 |
| Socio-economic status | 0.97 | 0.90 | 1.04 | 0.335 |
| BMI centered at 25 kg/m^2^ | 1.11 | 1.10 | 1.13 | < 0.001 |
| Physical activity (MET mins/week) | 0.91 | 0.85 | 0.97 | 0.007 |
| *Intercept* | 0.87 | 0.67 | 1.13 | 0.299 |

Linear mixed modelling results displayed; ^#^Trajectories based on 1333 participants; Reference group is Group-4 (Average-stable) with a membership of 40.8% (n=277); *Model results for BMI & WC based on 1242 observations representing 606 participants who attended one or more follow up. **Model results for hs-CRP based on 1139 observations representing 574 participants who attended one or more follow up.

**Supplementary Table-9: Head circumference (HC) growth trajectories *in-utero* and their association with BMI, WC & hs-CRP (Model 2)**

| **BMI (Model 2)*** | **Regression coefficient** | **95 % CI** | | **P-value** |
| --- | --- | --- | --- | --- |
| Trajectory Groups (*Ref- Group-4*) |  |  | | 0.415 (global) |
| Group-1 | -0.26 | -1.57 | 1.06 | 0.702 |
| Group-2 | -0.37 | -1.08 | 0.35 | 0.318 |
| Group-3 | 0.14 | -1.18 | 1.46 | 0.835 |
| Group-5 | -0.82 | -1.68 | 0.05 | 0.066 |
| Follow up Year (*Ref- Y20*) |  |  | | < 0.001 (global) |
| Y22 | 0.70 | 0.44 | 0.96 | < 0.001 |
| Y27 | 1.45 | 1.03 | 1.86 | < 0.001 |
| Sex- Males | -0.19 | -0.90 | 0.52 | 0.599 |
| Contraceptive use in females | -0.40 | -0.86 | 0.05 | 0.084 |
| Ethnicity | 1.26 | 0.26 | 2.26 | 0.013 |
| Socio-economic status | -0.59 | -0.94 | -0.25 | < 0.001 |
| Physical activity (MET mins/week) | -0.23 | -0.44 | -0.02 | 0.032 |
| Uncomplicated Hypertension | 1.18 | 0.29 | 2.06 | 0.009 |
| Maternal BMI at 16-weeks | 1.97 | 1.54 | 2.40 | < 0.001 |
| Maternal Alcohol drinking | -0.90 | -1.57 | -0.24 | 0.008 |
| *Intercept* | 24.10 | 22.98 | 25.22 | < 0.001 |
| **WC (Model 2)*** | **Regression coefficient** | **95 % CI** | | **P-value** |
| Trajectory Groups (*Ref- Group-4*) |  |  | | 0.515 (global) |
| Group-1 | -0.23 | -3.43 | 2.97 | 0.888 |
| Group-2 | -1.33 | -3.07 | 0.42 | 0.136 |
| Group-3 | **0.35** | -2.74 | **3.43** | 0.826 |
| Group-5 | -1.34 | -3.48 | 0.81 | 0.221 |
| Follow up Year (*Ref- Y20*) |  |  | | < 0.001 (global) |
| Y22 | 3.30 | 2.48 | 4.12 | < 0.001 |
| Y27 | 4.68 | 3.50 | 5.87 | < 0.001 |
| Sex- Males | 5.18 | 3.40 | 6.95 | < 0.001 |
| Contraceptive use in females | -1.73 | -3.20 | -0.25 | 0.022 |
| Ethnicity | 4.58 | 2.46 | 6.70 | < 0.001 |
| Socio-economic status | -1.62 | -2.41 | -0.84 | < 0.001 |
| Physical activity (MET mins/week) | -0.76 | -1.37 | -0.15 | 0.015 |
| Uncomplicated Hypertension | 2.31 | 0.30 | 4.32 | 0.024 |
| Maternal BMI at 16-weeks | 4.40 | 3.39 | 5.41 | < 0.001 |
| Maternal Alcohol drinking | -2.80 | -4.43 | -1.18 | 0.001 |
| *Intercept* | 75.63 | 73.01 | 78.24 | < 0.001 |
| **hs-CRP (exponentiated) (Model 2)**** | **Regression coefficient** | **95 % CI** | | **P-value** |
| Trajectory Groups (*Ref- Group-4*) |  |  | | **0.024 (global)** |
| Group-1 | 1.15 | 0.91 | 1.46 | 0.231 |
| Group-2 | 1.04 | 0.90 | 1.21 | 0.555 |
| Group-3 | 1.02 | 0.82 | 1.27 | 0.858 |
| **Group-5** | **0.78** | **0.65** | **0.94** | **0.008** |
| Follow up Year (*Ref- Y20*) |  |  | | < 0.001 (global) |
| Y22 | 0.82 | 0.70 | 0.96 | 0.011 |
| Y27 | 0.72 | 0.62 | 0.84 | < 0.001 |
| Sex- Males | 0.83 | 0.71 | 0.99 | 0.033 |
| Contraceptive use in females | 2.01 | 1.64 | 2.45 | < 0.001 |
| Ethnicity | 1.32 | 1.07 | 1.62 | 0.010 |
| Socio-economic status | 0.96 | 0.90 | 1.03 | 0.238 |
| BMI centered at 25 kg/m^2^ | 1.11 | 1.10 | 1.13 | < 0.001 |
| Physical activity (MET mins/week) | 0.90 | 0.84 | 0.97 | 0.005 |
| Maternal BMI at 16-weeks | 1.03 | 1.00 | 1.08 | 0.026 |
| *Intercept* | 0.87 | 0.67 | 1.13 | 0.307 |

Linear mixed modelling results displayed; ^#^Trajectories based on 1333 participants; Reference group is Group-4 (Average-stable) with a membership of 40.8% (n=277); *Model results for BMI & WC based on 1223 observations representing 596 participants who attended one or more follow up. **Model results for hs-CRP based on 1126 observations representing 566 participants who attended one or more follow up.

**Supplementary Table-10: Head Circumference (HC) growth trajectories *in-utero***

**and their association BMI in both sexes**

| ^#^Trajectories (Group membership) | Group-1  Low-stable  8.2%  (n=28) | Group-2  Average-falling  27.6%  (n=94) | Group-3  Low-rising  9.4%  (n=32) | Group-5  High-stable  12.9%  (n=44) | Global  P-value |
| --- | --- | --- | --- | --- | --- |
|  | **Males: BMI** | | | |  |
| Model 1 | -0.69 | 0.17 | -1.32 | 0.00 | 0.214 |
| Model 2 | -0.50 | -0.10 | -1.30 | -0.59 | 0.453 |
|  | | | | | |
| ^#^Trajectories (Group membership) | Group-1  Low-stable  8.0%  (n=26) | Group-2  Average-falling  31.5%  (n=120) | Group-3  Low-rising  8.1%  (n=23) | Group-5  High-stable  11.6%  (n=35) | Global  P-value |
|  | **Females: BMI** | | | |  |
| Model 1 | -0.33 | -0.29 | 2.52* | -1.31 | 0.033 |
| Model 2 | 0.22 | -0.73 | 1.77 | -0.94 | 0.079 |

Linear mixed modelling results displayed (β coefficient in kg/m^2^). Significant P-value: *<0.05

#Trajectories based on 1333 participants; Reference group is Group-4 (Average-stable) with a membership of 40.9% (n=133) for males and 42.7% (n=135) for females.

Model 1 covariates- age, ethnicity, SES & physical activity; Model 2 covariates- Model 1 plus Uncomplicated hypertension during pregnancy, maternal alcohol drinking, and maternal BMI at 16-weeks.

**Supplementary Table-11: BMI categories across Head Circumference (HC) trajectory groups**

**at 20, 22 and 27-years**

| Follow up | **Trajectories*** | Group-1  Low stable  (n=54) | Group-2  Average falling  (n=214) | Group-3  Low rising  (n=55) | **Group-4**  **Average-stable**  **(*Reference)**  **(n=277)** | Group-5  High-stable  (n=79) |
| --- | --- | --- | --- | --- | --- | --- |
| 20-years | **# of adults (N)** | **41** | **148** | **35** | **204** | **57** |
|  | <25 (kg/m^2^) | 33  (80.5) | 98  (66.2) | 22  (62.9) | 130  (63.7) | 38  (66.7) |
|  | Overweight | 3  (7.3) | 26  (17.6) | 9  (25.7) | 50  (24.5) | 11  (19.3) |
|  | Obese | 5  (12.2) | 24  (16.2) | 4  (11.4) | 24  (11.8) | 8  (14.0) |
| 22-years | **# of adults (N)** | **31** | **130** | **35** | **180** | **45** |
|  | <25 (kg/m^2^) | 24  (77.4) | 80  (61.5) | 22  (62.9) | 99  (55.0) | 32  (71.1) |
|  | Overweight | 2  (6.5) | 26  (20.0) | 7  (20.0) | 55  (30.6) | 8  (17.8) |
|  | Obese | 5  (16.1) | 24  (18.5) | 6  (17.1) | 26  (14.4) | 5  (11.1) |
| 27-years | **# of adults (N)** | **30** | **127** | **41** | **188** | **51** |
|  | <25 (kg/m^2^) | 16  (53.3) | 70  (55.1) | 20  (48.8) | 110  (58.5) | 31  (60.8) |
|  | Overweight | 9  (30.0) | 36  (28.4) | 14  (34.1) | 49  (26.1) | 11  (21.6) |
|  | Obese | 5  (16.7) | 21  (16.5) | 7  (17.1) | 29  (15.4) | 9  (17.6) |

Values represent frequencies with column percentages in parentheses *Trajectories based on 1333 participants, 679 of them analysed for BMI outcome; ^#^Reference group represents average growth; *BMI Categories: <25, Overweight (≥25 and <30), Obese (≥30) [in kg/m^2^]

**Supplementary Table-12: Birthweight and its association with BMI and WC**

| **Models** | **Regression coefficient** | **95 % CI** | | **P-value** |
| --- | --- | --- | --- | --- |
| **BMI (kg/m^2^)** | | | | |
| Model 1 | 1.61 | 0.79 | 2.42 | **0.001** |
| Model 2 | 0.85 | 0.17 | 1.53 | **0.014** |
| **Waist Circumference (cm)** | | | | |
| Model 1 | 4.62 | 2.66 | 6.58 | **<0.001** |
| Model 2 | 2.92 | 1.21 | 4.62 | **0.001** |

^*^Linear mixed modelling results; Results show change in BMI (kg/m^2^) or Waist circumference (cm) for every 1kg increase in birth weight; Model results based on 1242 observations representing 606 participants who attended one or more follow up. Model 1 adjusted for age, sex, ethnicity, SES and physical activity); Model 2-also adjusted for maternal covariates (Uncomplicated hypertension and maternal BMI at 16-weeks).
